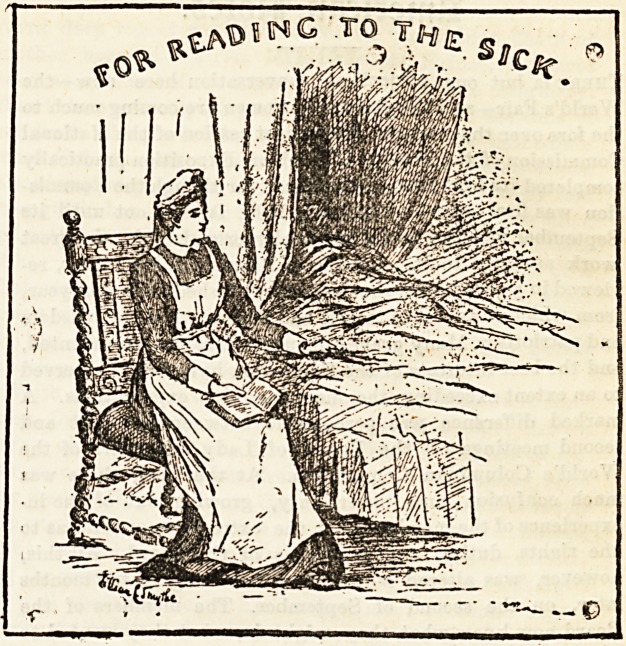# The Hospital Nursing Supplement

**Published:** 1891-10-17

**Authors:** 


### "The Hospital" Nursing Mirror.

Being the Extra Nursing Supplement of "The Hospital" Newspaper.

Contributions for this Supplement should be addressed to the Editor, The Hospital, 140, Strand, London, W.C., and should have the word "Nursing" plainly written in left-hand top corner of the envelope.

### En Passant.

UNIFORMS.—Grumbles come to us in answer to our note on pretty uniforms about several of the existing fashions. "I have just been to St. Thomas's to see a paying pupil there; the uniform is hideous; a flat spotted net cap, and a brown holland apron without a bib; for all the world like the dress of an ordinary charwoman." Then a country correspondent says: "Wigan has a fairly large hospital, well-fitted, with two children's wards. The sisters wear caps with strings tied under the chin—so ugly and uncomfortable-looking, and makes them hold their heads as though they had stiff necks." We must confess to a preference for white aprons with bibs, and mob caps without strings.

NURSES FOR THE MIDDLE CLASSES.—A Kentish Town nurse writes to say she would be very glad "between cases" to help those afflicted with sickness who are not rich enough to pay full fees and not poor enough to accept charity. The difficulty is to make the necessary arrangements. Our correspondent suggests that word should be left with the clergyman, but we think it would be wiser to appeal to the doctor. For instance, a doctor who has given a nurse a series of good cases could quite well say to her, "A gardener's daughter I am attending is very ill with pneumonia; will you go to her for 10s. a-week?" The doctor always has to attend many cases gratuitously, and many at reduced fees. Now that nurses are becoming more independent and taking their own earnings, they can afford to follow the example of their medical officer.

PAYING PUPILS IN ASYLUMS.—A new era in asylum life will be marked by the proposed departure at Berrywood (Northampton County Lunatic Asylum), of introducing a system of paying probationers. Some six or seven extra bed-rooms are being built, and when these are finished, for a small fee a lady will be able to receive not less than six months' training in mental nursing. We most heartily congratulate the enlightened authorities of Berrywood on being the pioneers in this matter. Of course, it yet remains to be seen if the scheme will succeed, but it certainly deserves to do so. It has long been difficult to secure as attendants on private patients women cultivated enough to be companionable in the patient's saner moods, and trained in mental nursing so as to be capable when the patient breaks out. Yet these attendants on private mental cases often receive £100 a-year. Doctors who take these cases into their own homes will surely approve the new scheme at Berrywood. Then as to the attendants now in asylums: it will lighten their work and introduce more variety to have these paying probationers. And surely the patients will rejoice in the presence of greater attention and more amusement provided by these extra nurses. Asylum work, be it mentioned, includes much that is ordinarily designated "menial," and it is to be hoped that none will attempt the life who is not used to sweeping, cleaning, &c., or at least ready to learn. The most likely probationers would be trained nurses, accustomed to discipline and routine, and ready to obey implicitly in return for this coveted experience amongst the insane. We all know how the whole tone of nursing was raised by the introduction of paying probationers; may the same occur in asylums.

SHORT ITEMS.—Miss Hart has resigned the editorship of the *Sanitary Record*.—Dr. H. R. Hutton gave an address on the objects of the Northern Workhouse Nursing Association, at the Poor-law Conference at Blackpool. In Lancashire there are fourteen workhouses, and in Cheshire five, without any trained nurses in them. A resolution in favour of the association was adopted.—A committee has been appointed to enquire into the nursing at Fulwood Workhouse.—We notice in the Slum Homes of the Salvation Army a card stating that "Nursing and Visiting Sisters" are ready to go to the sick. We hope these sisters are trained, for with all our admiration for the work done by the army, we object to the term "Nursing Sister" being used by any save a hospital-trained nurse.—Stafford Nursing Society reports 155 cases attended during the year.—Sister Catherine Gagnor, for over twenty years a nurse at the Monsall Fever Hospital, has been awarded a pension.

NURSES FOR STOCKPORT.—The Mayor of Stockport lately presided over a meeting for forming an association of district nurses for the town. The meeting considered the rules, which, after some discussion, were carried as follows: 1. That the name of the society shall be "The Stockport Sick-poor Nursing Institution." 2. The object of the Institution is to provide trained nurses for the sick poor at their own homes. 3. Subscribers of 5s. and upwards annually shall be members; and donors of £5 and upwards shall be life members of the Institution. 4. The Institution shall be managed by a committee consisting of twenty-one members to be elected at the annual meeting of the subscribers, five to form a quorum. This committee shall have power to elect such sub-committees as they may find necessary for the successful carrying out of the work. Three other rules dealing with arrangements for holding committee meetings, &c., were also carried. The surplus of the relief fund raised in January is to be devoted to starting this scheme.

THE ROW AT GLASGOW.—Unfortunately, Mr. McEwen has appeared in print in defence of the attacked party at the Glasgow Royal. The silent enquiry into evils, and disregard of virulent newspaper tirades, which the management showed at first, was most dignified and proper, and told greatly in their favour. Mr. McEwen's chief fault (in a letter which was mostly wisely written) was a hit at the resident medical officers, which has been well answered by the Senior House Physician, Dr. Browne, who says: "But with regard to our 'meddling with things we having nothing to do,' let me inform Mr. McEwen of a matter of which he is ignorant, in spite of his hobby to pay state visits to other hospitals, and that is that a large amount of the success of the treatment in this and other infirmaries—in other words, the welfare of the patients themselves—depends upon the way in which the patients are nursed and looked after, and if we are supplied with nurses tired out with overwork, or weak and unwell through faults connected with food, or feeling ill, yet cannot be relieved from duty on account of the paucity of the nursing staff, then I say that on selfish grounds alone we residents were justified in pushing this matter. And we had other well-marked grounds to go upon besides mere selfishness." We sincerely wish all parties would have the decency to wait for the official report.

### Lectures on Surgical Ward Work and Nursing.

By Alexander Miles, M.D. (Edin.), F.R.C.S.E.

Lecture XXXVII.—ARTERY FORCEPS.

(*a*) *Torsion Forceps* are employed to seize and then to twist arteries. This method of arresting hæmorrhage was first prominently advocated by Mr. Bryant, who has employed it very largely in his practice. The forceps have flat parallel finely-serrated blades, secured by a sliding catch; they are applied either in the line of the divided vessel, or, better, at right angles to it, and then turned five or six times round, so as to rupture the inner coat of the vessel, and so arrest the flow of blood. They should be left on for a few minutes after twisting till the clot has fairly formed inside the vessel.

(*b*) *Liston's Catch Forceps* in shape resemble ordinary dissecting forceps, but they are furnished at the point with sharp teeth, and in the middle with a spring catch, by means of which they retain their hold on a vessel, and may be left for some time. The ligature should be applied well above the point at which the vessel is seized, especially if it be a large trunk, as bleeding is apt to take place from the wound in the vessel-wall made by the forceps.

A modification of these forceps by Wakley has the blades broadened out and fenestrated, so that the point becomes conical, and facilitates the application of the ligature.

(*c*) *Spencer Wells' Forci-pressure Forceps* are perhaps the most generally used instruments for the arrest of hæmorrhage. They have the advantage of being very readily applied, and of holding securely even large vessels. If left on for a few minutes the pressure exerted by them arrests bleeding from smaller arteries, or if this does not occur, then they may be used as torsion forceps, or a ligature may be applied according to the taste of the surgeon.

They differ from the pattern used by Pean, in having the blades inseparable, and the joint only a short distance from the point.

(*d*) *Pean's Forci-pressure Forceps* differ from those of Sir Spencer Wells only in having the blades separable (for purposes of cleanliness), and in having the joint about half-way down the blade. These forceps are more apt to jump off the vessel when left hanging than those of Wells', otherwise they are equally good.

(*e*) Various forms of small portable instruments have been devised for applying to small bleeding vessels, but are not much used, especially in hospital. Of these may be mentioned (1) the *serre-fine*, or twisted wire forceps, chiefly used in France; (2) *Dieffenbach's Bull-dog Forceps*; and (3) *Maw's Artery Forceps*.

(*f*) Of the older instruments used in the arrest of hæmorrhage may be mentioned (1), the *Tenaculum*, a sharp hooked instrument with a wide curve. The bleeding artery was transfixed by this and pulled out of its bed while a ligature was applied.

(2) *Asalini's Forceps*, which is really sort of double tenaculum, acting with a spring. It is seldom or never used now.

The illustrations are used by kind permission of Messrs. J. Weiss and Sons and Messrs. Maw, Son, and Thompson.

### The Nurses' Bookshelf.

### THE HOSPITAL ANNUAL.*

The "Annual" for 1891-2 is just out, and contains statistics of all the hospitals and kindred institutions of the United Kingdom, besides particulars of the chief hospitals of the colonies, and chapters on various subjects. Here are full directions as to "How to Become a Nurse," with particulars of the training given at different schools; here are articles on "Monthly and Army Nursing," on "Nursing Abroad," on "Dispensing," on "The Pension Fund," on "Massage"; in fact, on all those points which our querists are perpetually asking us about. There are also terrible-looking tables about cost per bed and cost per head, which, we suppose, are of great interest to secretaries and committees; and there is a chapter on book-keeping and accounts, which would be invaluable to the Matron of a cottage hospital. Then here are the rules of all the convalescent homes and institutions for the lame, the halt, and the blind—all those difficult cases which trouble our correspondents so much. One and all, we recommend them to procure the "Annual," and promise them they will find a most useful addition to their reference library.

### A MANUAL FOR MOTHERS.†

This is a well-written book, and likely to be useful to nurses and district visitors, as well as to mothers. The chapters on preventable disease and infection are really excellent. In the preface, Dr. Joll states that it is on the subjects of "sanitary and preventive medicine." He has tried to break new ground and give information which other manuals lack. This book ought to have an index, and ought, as every book about infants should, to have some warning as to the ease with which children can be suffocated. With ten deaths weekly in London from suffocation, it seems extraordinary how seldom mothers and nurses are warned that a head flannel or shawl over an infant's face may cause its death. Otherwise, we can find no points to criticise.

### A HINT FOR BAZAARS.‡

Charities of all kinds profit by bazaars, and we know of no more successful addition to such an entertainment than a Fine Art Gallery arranged on the lines of this pamphlet. Here we have "View of the Needles," by A Sharp (exhibited a packet of needles); "The First Letter" (letter A), "A Pair of Black Eyes" (black eyes for a dress), and so on. Those who are going to hold a bazaar had better buy this pamphlet.

* "Burdett's Hospital Annual." Published by The Hospital (Limited). Price 3s. 6d.

† "Nursery Hygiene." By Dr. B. Barnett Joll. (Kimpton.) Price 2s. 6d.

‡ "The B zaar Fine Art Gallery." By E. W. Webb. French. Price 1s.

### Notice.

Friedenheim.—A sale of work on behalf of Miss Davidson's Home for the Dying, Mildmay Road, will be held on Thursday and Friday, October 22nd and 23rd, at Hensleigh, 36, Pyrland Road, N. Open from eleven a.m. to eight p.m. Friends heartily welcome, and contributions of work, &c., will be gratefully received by Miss Brook, at the above address.

### The Cyprus Society.

A word about the Moslem schools in the island. Ninety-nine of these receive aid from Government, two of these being girls' schools, 19 temporarily closed; 92 teachers employed, only two of them mistresses. Average annual salary, £18 18s. Number of children enrolled in 80 schools (i.e., omitting 19 closed), 2,012 boys and 597 girls. Average enrolment for each school, 32; average daily attendance, 25. The annual average cost of a Moslem elementary school (£20 13s. 3d.) is barely more than half the cost of a Christian school. And the whole amount required for these 80 Moslem schools comes from three sources, and in these proportions:—Government grant, 45 per cent.; endowments, 21½ per cent.; voluntary contributions, 33½ per cent. There are 12 Moslem schools which receive no aid from Government. These, presumably, are better off, financially, than the 80 schools of which particulars have been given. True, some of them may have been refused any aid on account of more than ordinary deficiency in their work; still, as they remained open they must have had funds from some source. Aided or unaided, there are few Moslem schools in which any systematic instruction is given, the more remarkable as any exceptional school here or there striking out a better line of work is immediately rewarded with additional pupils. Mr. Spencer seems to think that of all the Moslem schools only two can be mentioned as actually improving and giving hope of still better things by-and-bye.

We have placed these figures before our readers, thinking the particulars interesting in themselves, and a distinct addition to the information which most people possess about our fellow-citizens in the island of Cyprus. But we have also had another object in view. What better argument for the educational efforts of the Cyprus Society than these plain facts now detailed? What could show more convincingly that the reforms are great and urgent in which this society leads the way, and asks us to unite? At Lambeth Palace, the Primate of all England assured the Archbishop of Cyprus "that, not only the Church, but the whole of the people of this country, had the deepest feeling of responsibility towards the noble island, and the strongest desire that it should be rendered most suitable as a home for its people, representing so many races and so many ages." None will be disposed, we imagine, to question this statement of the claims of Cyprus upon the English people; and the Cyprus Society exists to-day, both as the acknowledgment of a responsibility, and as the instrument by which this responsibility may be effectively discharged. It is proposed to raise £300 a-year for the maintenance at Cyprus of two clergymen, competent educationalists, to inaugurate, develop, and superintend better plans of education, and to train up native teachers. The hon. secretary (30a, Wimpole Street, W.) will be ready to give the fullest information on this or other parts of the society's programme to any who are willing to assist in the work.

### A Bed for a Sick Nurse.

We had by Tuesday received the following guinea subscriptions, which we acknowledge with thanks: (1) Nurse C. J. Bennett, (2) H. M., (3) Miss Pritchard, (4) Miss Yorke, (5) The Committee of the Macclesfield Infirmary, (6) Nurse W. P., (7) Mrs. Goslett; and the following smaller sums: One of the First Thousand, 6d.; A Nurse, 1s.; Some of the Nurses, Burton on-Trent Institution, 6s.; and a Private Nurse, 5s. We have the promise of one more guinea subscription, making eight in all, but we hope for more as the days go by. We have, dear nurses, quite given in as regards our plan of collecting the thirty guineas entirely from our non-nurse readers; if you will respond so rapidly and so readily why it must e'en be so; and may none of you miss these shillings you are giving up for the good of your sick or weary sisters.

### THE CLOUD.

We have gone on for some years cheerfully and well, taking our daily food which has nourished and supported us, when gradually a cloud seems to have overshadowed our lives, and we have become thin and pale, and sad, for nothing that we eat agrees with us. Now any diseases which attack the digestive organs, that is which prevent the taking or retaining food, are very depressing, and when we have lost the power to do so we begin to see that what we have not looked on as a mercy at all, has been a really constant manifestation of God's power and goodness, which has given us day by day our daily bread and caused us to enjoy it. We think it a strange thing now it is otherwise, an accident speedily to be got rid of. This illness resembles a cloud, as do many other sharper complaints, for they shut us out from pleasure and prevent our enjoying the society of our friends even when we are not entirely laid by. But is the cloud all dark? Do we fear that it will burst suddenly upon us and overhelm us? Be not afraid the darkest cloud has a silver lining; we shall see it if we look for it.

Our heavenly Father knows perhaps that we took our good health as our right, a state from which our goings would not side, or as a common mercy for which we are not even grateful. As when a storm is approaching we look out for shelter, so now in our sickness we should seek Him who is a shelter from the wind, and a refuge from the storm. In His protecting care we can rest safely, looking beneath the darkness which surrounds us and seeing the bright light coming straight down from His glorious presence. This hiding of Himself, then, will make us value our mercies and teach us to be grateful for all the blessings of His hand. For the many days and months and years that we have taken our food as a right, let us now set our hearts to thanking God in earnest. Gratitude brings cheerfulness, and see the change; lo! the silver lining is beginning to show itself. All those about us appear kind and sympathetic now that we have grateful hearts, and by these earthly steps we mount to Heaven. And the light will gradually increase till the sky is full of glory. We have watched the moon rise on a cloudy night, at first she is only visible at intervals, through the rifts in the clouds; presently she is entirely hidden by a blacker one, but gradually it has a lovely fringe of light, and soon the beautiful orb comes forth in all her glory; the clouds vanish, and she sails like a conqueror through the night, mistress of the sky. So we may find our clouds of sickness and sorrow disappear if we borrow our cheerfulness from the Sun of Righteousness.

### American Notes.

There is but one subject of conversation here now—the World's Fair—and the American women are coming much to the fore over the matter. The recent session of the National Commission of the World's Columbian Exposition practically completed its first year of existence, for though the Commission was first assembled in June, 1890, it was not until its September meeting that it was really organized for the great work assigned to it. President Palmer, in his report, reviewed in a general way the work accomplished during the year, from which it appeared that it had been marked by wisdom and patriotism. Many grave difficulties have been surmounted, and the best interests of the Exposition have been subserved to an extent exceeding the most sanguine expectations. A marked difference was perceptible between the first and second meetings of the Board of Lady Managers of the World's Columbian Commission. At the first, there was much confusion and uncertainty, growing out of the inexperience of the members and the extreme vagueness as to the rights, duties, and privileges of the Board. All this, however, was altered when it convened again ten months later, on the second of September. The members of the Board now knew what they might do, what they wanted to do, and how to set about it. In the main gallery will be a special exhibit of the most brilliant things women have done. Precisely what these will be it is, of course, impossible now to foresee, since the whole world is to be drawn upon for the supply. But the value of the showing is ensured by the high standard established by the recent action of the Board, which resolved "to let no sentimental sympathy for women induce the acceptance of mediocre work." In the smaller rooms will be model home rooms, and displays of a philanthropic character. The Bellevue Hospital of New York will probably—as the finest institution of the kind in the country—have charge of the trained nurse department. Model kindergartens will be a conspicuous feature. Lady Aberdeen is taking great interest in the arrangements.

I send the sessional report of the Plymouth Church, Indianopolis, to show how light and culture are circulated by those young churches which have so much more spring and go than yours in England. You will see that there is a study class in social science, conducted by Mr. Alexander Johnson, who is the Secretary of the Board of State Charities. He was invited to give a series of lectures before the Chicago Institute. He has remodelled them, and will give them before the Plymouth Institute. The subject is one of the greatest importance. The growing number of the insane, deaf, dumb, blind, feeble-minded criminals, paupers, neglected children, and the poor, make it necessary that we should give thoughtful consideration to their care and treatment; preparatory to the different lessons, the class visits the hospitals and institutions which are trying to remedy the evil under discussion.

Here are some particulars of the New York Hospital Training School. A class is started each April and each October with the expectation of getting fifteen competent pupils out of each class. The course lasts for two years, and usually the class in the beginning consists of from forty to fifty young women on probation. When the probationers are weeded out, at the end of a month or so, the fittest only have survived, and about sixty per cent. have been found to be undesirable. Candidates having been accepted on probation are given free board and washing, but no pay, and are put to work in performing minor duties of assistance in the various wards. Some find that the work does not suit them, and the directress finds that others are not suitable for the work. Before the end of two months has passed the class has been formed, and the accepted women don the cap, dress, and apron of the hospital, and become junior assistants, with a salary of ten dollars a month. The senior assistants get thirteen dollars a month, and have charge of the wards during the night.

The second report of the John Hopkins Hospital deserves to be given in full if space only permitted it; but failing such space here are the chief items of news to be gathered from it. In March and October, 1890, classes of eleven pupils were selected from the probationers and commenced their training. Very numerous lectures were given on The Hygiene of Apartment, Gynæcology, Children, Dietetics, Massage, &c., besides the usual lectures on Nursing. The lecture courses in America are far fuller than in England. In June the school undertook the nursing of the children at the Thomas Wilson Sanitarium, supplying a head nurse and three pupils. Miss Hampton continues in charge of the Training School, and has lately returned from visiting England, where she inspected the chief hospitals. During the year a cooking school has been organised, and each pupil has attended it for three weeks, and learnt the method of preparing about 150 different articles of sick diet; the course was followed by an examination, when such questions as "Why is bread, improperly toasted, unwholesome?" were asked. The nurses have started a "Journal Club," for the study of current literature on nursing, a proceeding which other probationers interested in their profession might well follow. It is hoped in the present year to start a district nurse to attend to the out-patients of the hospital.

And speaking of current literature on nursing, let me recommend to your readers Dr. Sarah Post's "Massage Primer," published at 13, W. 42nd Street, New York, price one dollar. This is not a compilation of cases, but consists of solid instruction arranged for use as a text-book in three parts. The first is devoted to a description of the motions of massage, giving the various stroking, kneading, and percussing motions. The second is devoted to general massage, giving the minuter details in regard to the management of the patient, length of application, the peculiarities of patients, references to the Weir Mitchell and Playfair treatment, &c. The third is devoted to local massage, embracing synopsis of the literature of the subject, including dosage and frequency of repetition required to effect cure. Among the subjects treated are massage of the neck, breast, abdomen, massage for writer's cramp, and pelvic massage, including massage of the coccyx. The illustrations are splendid, and are reproduced from photographs; this secures their absolute correctness, and gives them a great advantage over all other illustrations I have seen. Instantaneous photography might be much more widely used for nursing and medical illustrations. Also let me recommend to nurses in children's wards the sixpenny monthly magazine called "Babyhood," which can be obtained at 93 Fleet Street, London, though it is really an American publication. The late numbers have held excellent articles on "Diphtheria," by Dr. Lewis Smith, "Eczema," by Dr. G. T. Jackson; "Hay Fever," by Dr. S. Ashurst, and plenty of annotations on minor subjects, and answers to pertinent queries respecting the care, diet, and clothing of children in sickness and in health. For instance, in a note on sterilized milk, the author says: "Now, the study of bacteriology among its many results brought out prominently two facts regarding milk. One was that milk could accidentally contain various kinds of germs. The other fact was that the process of sterilization could be applied to milk in its fresh state and without condensation, and that such sterlized milk might be indefinitely kept without decomposition. Boiling of drinking water is believed to remove from it the danger of its conveying typhoid fever, for instance; boiling or sterilising of milk is believed to do the same for it."

### Everybody's Opinion.

[*Correspondence on all subjects is invited, but we cannot in any way be responsible for the opinions expressed by our correspondents. No communications can be entertained if the name and address of the correspondent is not given, or unless one side of the paper only be written on.*]

### NURSES' FOOD.

"An Old Nurse" writes: "May I say that my experience agrees with your correspondent, "L.," and I cannot but think nurses nowadays are losing that *esprit de corps* which enabled them formerly to put up with the little roughnesses unavoidable to hospital life? I have been many years in the work, and I notice year by year how these complaints increase, although nurses are certainly far better off than they were formerly. I was trained in a county hospital, where most of the nurses were ladies, and I remember very little grumbling, though the food was plain, joints and milk pudding for dinner, and only bread and butter for breakfast. Now I am Matron of a hospital with only a small staff of nurses, which, of course, makes it easier to give them plenty of variety, and their meals are as follow: First breakfast, tea and bread and butter, before going into wards; eight o'clock, second breakfast, tea or coffee, bacon, fish or eggs, and marmalade; eleven, lunch, milk and bread and butter; two, dinner, roast meat, pies, or stew, two vegetables, pudding or tarts, often fish or soup; half-past four, tea, always jam, often hot cakes; eight, supper, meat (hot or cold), pudding, soup, or porridge, cheese, and jam. I think you will agree with me this is a most sufficient dietary, and with management it is not extravagant, and yet there are constant grumbles over the merest trifles, and from nurses who are by no means ladies. I believe if nurses would think a little less of themselves, and realise more fully the highness of their vocation, we should hear less of these trivial complaints, and if nurses would only go to their Matron in a friendly way, and ask her to try and improve matters, and even lay it before the Committee for them if needful, I am certain they would find any reasonable wishes would be granted. P.S.—I hear people outside say that "private nurses are so tiresome about their food." All this is very disgusting to noble-minded women.

### MALE NURSES.

"One of Them" writes: One fails to wonder at your inclination to stricture male nurses in general for the fault of one who has so far forgotten himself as to write in "Harper's" what you very justly condemn. The style and composition of the sample given, *en passant*, needs no comment. Believe me, gentlemen, the writer in "Harper's" should not be taken as a specimen of a well-trained male nurse, hence, I plead that we lose not your valuable advocacy and powerful aid in any future plans, or discussion that may arise, aiming at raising male nursing to its higher level, or, in weeding out what is objectionable, and the chief cause of the distrust you truly tell us many doctors regard us with. While thanking you for aid already given, I respectfully offer this refutation, confident that it expresses the sentiments of the many.

### Presentation.

On September 28th the nursing staff of Monsall Hospital presented Miss Florence M. Calvert (Lady Superintendent) with a handsome gilt clock—having a suitable inscription—on the occasion of her leaving the hospital for the Manchester Royal Infirmary. Miss Calvert's resignation has caused deep regret amongst the members of her nursing staff, for Monsall has to thank Miss Calvert for numerous improvements in its nursing and general management. We have no doubt that Miss Calvert will have an opportunity of displaying her managing powers, even in such a well-organised hospital as the Manchester Royal Infirmary.

### Death in Our Ranks.

With deep regret we have to record the sudden death of another hospital Matron, Miss Charlotte Prince, for three years at the head of the nurses of the Blackburn Infirmary. Miss Prince was only 43 years of age, and was at the time of her death taking a holiday at Bournemouth; the immediate cause of death was ænemia, coupled with general weakness. A contemporary writing of Miss Prince says: "Sauvity and strength in her were blended, and none but those who came in immediate contact with her could form any proper estimate of her zeal, or value at its worth the sweet radiance of a character that in scenes of trying duty had learnt the great lesson of self-renunciation for the good of others and preached the highest Christianity by the practice of it:" Miss Debell is acting as Matron at Blackburn for the present.

With deep regret we hear of the death of Miss Jane Harrison Orr, Honorary Lady Superintendent of the district nurses of Ballymena, Co. Antrim. Miss Orr and one of her nurses contracted typhus from a case in the district; Miss Orr died at Hugomount on October 6th, and the nurse is not expected to recover. About nine years ago Miss Orr trained at St. Bartholomew's, especially to qualify herself for attending the poor round her own home, a duty she has discharged so well that her death has caused most profound regret amongst all classes of society in the north of Ireland. To her fellow-labourers in the nursing world she has bequeathed a priceless legacy of influence and example.

At Greenwich, on Monday, an inquest was held as to the death of Jeanette Ferrier, who was found lying unconscious in the gateway of a house at Mazehill on Monday week, and died in the infirmary a few hours afterwards. The deceased had at one time been Matron of the Hospital for Children and Women, Waterloo Road. The inquest was adjourned.

### Appointment.

Inverness Infirmary.—Miss Macconnachie, now of Dufftown Hospital, and who trained at Glasgow Western Infirmary, has been appointed Matron at Inverness in place of the late Miss Falconer. The nurse who acted so efficiently as Matron during the vacancy has been awarded a gratuity.

### Notes and Queries.

(1) Can anyone (from practical experience, if possible) inform "Enquirer" what staff, including servant, &c., is required for the working of a cottage hospital with eight or nine beds, and a private ward, and where the washing is done in the house?—*E. T.*

(2) Can any nurse tell me of anything to keep away fleas? Camphor has been tried without effect.—*E. T.*

(3) Would a nurse requiring rest join another in apartments at Bournemouth early next month?—*C.E.*

(4) *The Jews and Cancer.*—Is it known whether the Jews are as subject to cancer as others who are in the habit of consuming swine-flesh?—*Margaret.*

### Answers.

*M. S. R.*—Your query is vague. It is usual to pay a nurse her fee at the end of a case, and she has then a right to it. She can take proceedings in the county court to secure the payment if there is any difficulty.

*E. M. W.*—Socks received for No. 2 competition.

*Sister Mona.*—Thanks; but we shall not mention that lady's name again, and are sorry we ever did so. The last reports were most unsatisfactory, and there is no business-like account of the funds.

*Poor Pro.*—A teaspoonful of powdered borax and a teaspoonful of glycerine in half a tumbler of water will make the mouth wash you want.

In answer to W. Rivers' questions as to the best antipyretics and hypnotics to take abroad, we consider that Quinine and its compound preparation, Warburg's Tincture to be the best general antipyretics; at the same time a supply of antipyrin or phenacetin should be taken. With regard to hypnotics, sulphonal for general insomnia without pain is good, also chloramide, but some preparation of opium should also be carried, as it is often absolutely necessary, and there are many cases where pure hypnotics, as the above, are useless. Burroughs and Wellcome's preparations are excellent.

*Rosa, Nurse L., and others.*—Many letters are unavoidably held over this week.

*Nurse Alice.*—See the list of medical officers to the Children's Hospital in the town you name.

*Nurse G.*—Your contribution to competition 4 safely received.

### From Pharmacy to Farming.

Joshua Harman, sheepdealer and fellmonger, of Knap Hill West, one mile from Hook-in-the-Vale, was said to be wealthy and a miser. He was a "close" man concerning his private affairs, but he had once been heard to say that he placed no confidence in banks. And so it was reported in Hook that Joshua hid his wealth in his house, and slept with a charged muzzle-loader over his pillow. Some gossips estimated his hoard at thousands; others at hundreds of pounds. Yet Knap Hill was almost a ruin; the flooring was warped and worm-eaten, the roof let the rain in, and a musty, mousey smell pervaded the building.

Still, the old house, half-concealed by a clump of beech trees, was a picturesque landmark from the Marley-road, and in spring the bloom of the orchard shone white before the dark slope of heathy hills above the vale. A slow, deep stream, edged with polled willows, flowed between the house and the high road, and the meadows on either side were dotted with the farmer's sheep.

One June morning, the fellmonger was riding down the lane that leads to the high road. The unkempt white pony, blind in the left eye, cautiously crossed its fore-legs, and Joshua rose now and then in the stirrups to peep over the hedge at the four men who were mowing in Biddle's Piece. Halfway down the hill the Hook, postman met him, and gave him a letter.

"From Master Ed'ard," chirped the old letter carrier. "Leastways, I guess so by the post-mark. And how's the young man gettin' on in Lunnon? A promisin' lad, he wur, when he served 'prentice in Marley."

Joshua pushed his forefinger under the flap of the envelope, and read the letter with a puckered forehead, and an uneasy expression of the eyes.

"You ain't very spry to get on wi' your letters, Hankey," said Joshua, who hated inquisitive gossips. "The squire'll be arter you if you keeps him waitin'."

"You be a short-spoken 'un," muttered Hankey, as the fellmonger dug his heel into the pony's side, and trotted away to avoid questionings.

During the morning Joshua was too busy to give much thought to the letter in his pocket, but after his frugal dinner of cold mutton and thin ale, he re-read it through a pair of silver-rimmed spectacles. Mrs. Powell, the housekeeper, a second cousin of Harman's, was cleaning the table. Holding the letter at arm's length, and twisting round in his chair, Joshua tapped the sheet with his spectacle case, and said :—

"It's a pretty pass, Martha, when youngsters invite theirselves! Here's Edward coming down to spend a day or so wi' us. Can we make up a bed?"

"Comin' to-morrow?" ejaculated Mrs. Powell, putting down the tray with a clatter.

"Yes; short notice and rum manners, eh?"

"We've only one sheet to spare," she said. "I don't count on visitors."

"Nor me," returned Joshua. "*I* never gave folk the trouble o' puttin' theirselves out to find *me* board and vittels for a week. Well, he must put up wi' what we've got, and take pot-luck."

At noon the next day a slim, pale young man of three-and-twenty, carrying a small leather trunk, got out of the carrier's cart at the end of the lane. He came slowly up the hill, often turning to rest and to look at the beautiful valley. His eyes, long accustomed to the gloom of the shop corner in London, where he compounded prescriptions, were dazed with the glare of the sunshine, the gleam of the rippling stream, and the vivid hue of a smokeless, cloudless sky. Never before had the country seemed so vernal, so like a dream. Every sight and sound was new, delightful, and full of strange interest. For two years he had followed a distasteful occupation in the din of a City street, with never a peep of green fields; and the smell of drugs and toilet soaps still lingered in his nostrils, in spite of the mingled scents of new-mown grass and honeysuckle.

Mrs. Powell met the visitor at the door, and held out a yellow hand, as she asked if he had dined.

"I had a snack in Marley, thank you," said Edward, "but if you've a glass of cider, I shall be glad of it. It's hot walking up that hill."

"We've no cider, Mr. Edward, and I don't think there's more'n a pint of ale in the cask. You see, your uncle don't lay in much stock, 'cause ale don't keep brisk this weather. I might 'a' given you some milk, but barring what I've saved for tea, it's all gone to Marley."

"Then I'll be content with water," said Edward.

He sat down, and looked around the shabby parlour. The carpet was threadbare and patternless, the springless sofa was rent in the cover, and showed the horsehair in two places, and the mantelshelf was littered with paper spills, bits of ruddle for marking sheep, balls of string, and a pair of horse-clippers. When Joshua came into the house he was in no amiable mood. The men in Biddle's had petitioned for more beer.

"'Taint as though they aint well paid," he said. "But there, do your best by your farm hands, and they'll grumble. Well, Master Edward, how's the chemisting going?"

"I'm heartily tired of the trade, Uncle Josh," sighed the young man. "I've come down to consult you about going into the veterinary profession."

Joshua screwed up his mouth, and opened his cold grey eyes.

"I've heard say that medicines bring in all profit, or pretty nigh so," said the fellmonger. "If 'tis true, what in the world do you want to change your trade for? If you have the chance to pass your examinings for a vet., you're still no better off nor you be now. You've got to find a practice, and meantimes how're you going to get clothes and vittels? That's the point, Master Edward."

(*To be continued.*)

### Amusements and Relaxation.

### SPECIAL NOTICE TO CORRESPONDENTS.

**Fourth Quarterly Word Competition commenced October 3rd, ends December 26th, 1891.**

Competitors can enter for *all* quarterly competitions, but no competitor can take more than *one first prize* or *two* prizes of any kind during the year.

Proper names, abbreviations, foreign words, words of less than four letters, and repetitions are barred; plurals, and past and present participles of verbs, are allowed. Nuttall's *Standard* dictionary only to be used.

The word for dissection for this, the THIRD week of the quarter, being

"IRELAND."

| Names. | Oct. 8th. | Totals. | Names. | Oct. 8th. | Totals. |
|---|---|---|---|---|---|
| Lightowlers | 7 | 7 | Nurse J. S. | 6 | 6 |
| Bonne | 7 | 7 | Jenny Wren | 6 | 6 |
| Morico | 7 | 7 | Darlington | 6 | 6 |
| Robes | 7 | 7 | Nurse G. P. | 6 | 6 |
| Dulcamara | 7 | 7 | Hetty | 5 | 5 |
| Psyche | 7 | 7 | Janet | 3 | 3 |
| Agamemnon | 7 | 7 | | | |

### Notice to Correspondents.

All letters referring to this page which do not arrive at **140, Strand, London, W.C.**, by the *first post on Thursdays*, and are not addressed PRIZE EDITOR, will in future be disqualified and disregarded.

N.B.—Each paper must be signed by the author with his or her real name and address. A *nom de plume* may be added if the writer does not desire to be referred to by us by his real name. In the case of all prize-winners, however, the real name and address will be published.

## Figures and Tables

**Figure f1:**
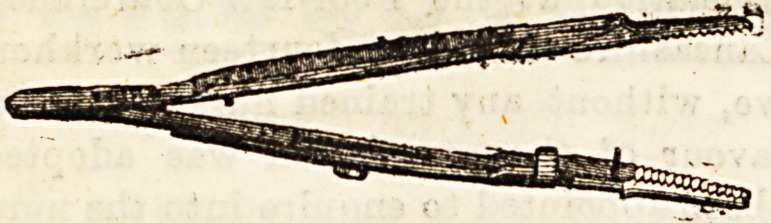


**Figure f2:**
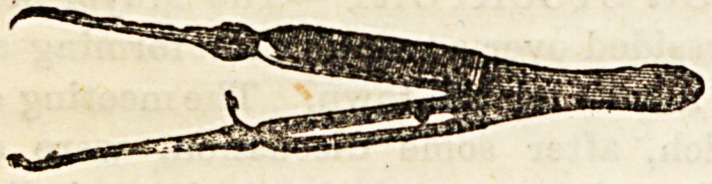


**Figure f3:**
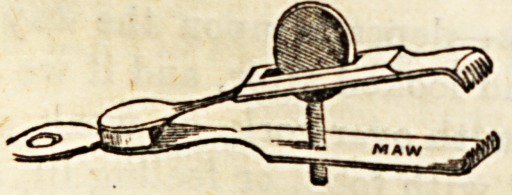


**Figure f4:**
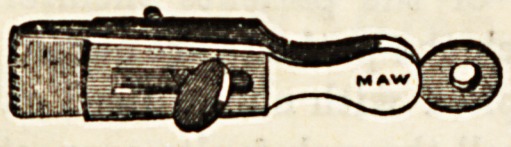


**Figure f5:**
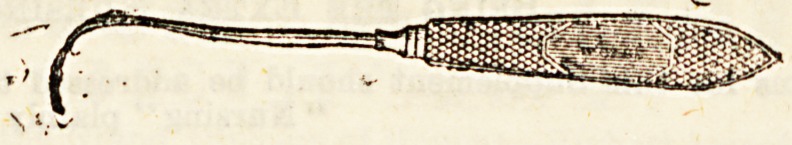


**Figure f6:**